# Extrinsic cell death pathway plasticity: a driver of clonal evolution in cancer?

**DOI:** 10.1038/s41420-022-01251-7

**Published:** 2022-11-26

**Authors:** Eric Seidel, Silvia von Karstedt

**Affiliations:** 1 University of Cologne, Faculty of Medicine and University Hospital Cologne, Department of Translational Genomics, Cologne, Germany; 2 CECAD Cluster of Excellence, University of Cologne, Cologne, Germany; 3 University of Cologne, Faculty of Medicine and University Hospital Cologne, Center for Molecular Medicine Cologne (CMMC), Cologne, Germany

**Keywords:** Tumour heterogeneity, Cell death

## Abstract

Human cancers are known to adhere to basic evolutionary principles. During their journey from early transformation to metastatic disease, cancer cell populations have proven to be remarkably adaptive to different forms of intra- and extracellular selective pressure, including nutrient scarcity, oxidative stress, and anti-cancer immunity. Adaption may be achieved via the expansion of clones bearing driver mutations that optimize cellular fitness in response to the specific selective scenario, e.g., mutations facilitating evasion of cell death, immune evasion or increased proliferation despite growth suppression, all of which constitute well-established hallmarks of cancer. While great progress concerning the prevention, diagnosis and treatment of clinically apparent disease has been made over the last 50 years, the mechanisms underlying cellular adaption under selective pressure via the immune system during early carcinogenesis and its influence on cancer cell fate or disease severity remain to be clarified. For instance, evasion of cell death is generally accepted as a hallmark of cancer, yet recent decades have revealed that the extrinsic cell death machinery triggered by immune effector cells is composed of an astonishingly complex network of interacting—and sometimes compensating—modes of cell death, whose role in selective processes during early carcinogenesis remains obscure. Based upon recent advances in cell death research, here we propose a concept of cell death pathway plasticity in time shaping cancer evolution prior to treatment in an effort to offer new perspectives on how cancer cell fate may be determined by cell death pathway plasticity during early carcinogenesis.

## Facts

- Cancers adapt under selection pressure imposed by treatment
- Functionality of extrinsic cell death pathways is frequently lost in cancer
- Cancers lose extrinsic cell death pathways as a consequence of selection pressure imposed by adaptive immunity.

## Open questions

- What are the mechanisms of survival-of the-fittest during early carcinogenesis?
- Which cell death pathways are lost in cancer?
- Is there a hierarchical sequence to switching off extrinsically-triggered regulated cell death pathways during early cancer evolution?

## Darwinian principles of somatic evolution in cancer

Since Charles Darwin’s work on the origin of species, the pursuit of a deeper understanding of the diversification of life has always demanded a dual approach. Striving to understand the “how” of evolution has led to great efforts in clarifying the evolutionary history of certain species. Considering the “why”, on the other hand, has led to the definition of underlying principles, most famously natural selection, which, in turn, has ultimately yielded a deeper understanding of the molecular genetics of life. Natural selection essentially describes a process, by which the distribution of a variable, inherited trait may change within a population over the course of generations in adaption to selective pressure. Building on early insights into the biochemical, genetic, and immunological nature of neoplasms, PC Nowell in 1976 pioneered the idea of neoplasm evolution by proposing that the same principles underlying the evolution of multicellular species should also apply to neoplasm formation [1]. Since then, advances in the field of oncology have, in fact, substantiated both, Nowell’s claims and our understanding of the “how” and “why” of neoplasm formation, a process that has gathered even further momentum with the advent of next-generation sequencing, multi-regional sampling and multi-omics approaches during the last decade [2–4]. It is now widely accepted, that cancers fulfill all necessary requirements for the selection-of the-fittest [5]. Common cancers are genetically heterogenous entities [6–9], and genetic variation has been implicated in tumor evolution [10]. Variation is largely caused by genome instability, a well-recognized feature of many, if not all, cancers [11]. Furthermore, tumor development and progression seem to evolve through the inheritance of certain driver mutations that arise early on during tumor evolution [12]. Many tumors comprise cells of clonal origin [13–16], and cells harboring apoptosis-inactivating mutations, e.g., in TP53, may expand and overgrow other subpopulations in different entities [17–19]. Strikingly, the sequence of events influencing individual clonal fitness may impact tumor outcome, as a study in a KRAS^G12D^-driven genetically engineered mouse model of colorectal cancer has shown that differently timed sequential combinations of targeted knockout of *Apc*, *Tgfrb2*, and *Trp53* may greatly impact tumor onset, stage, and the number of metastasis [20]. Finally, tumor cells, just like a population of any given species, hold the ability to adapt under selective pressure, besides the loss of antigen presentation also through modulating cell death pathway availability, a concept discussed in this article.

## Cell death in cancer evolution: not just post-treatment

Adaption of cancer cell populations by mitigating cell death is best exemplified by the occurrence of drug resistance during cancer treatment, which is essentially as old as modern chemotherapy itself [21]. Drug resistance in cancer may occur via a plethora of mechanisms, including increased efflux, decreased drug uptake, drug inactivation, and active export of the drug from cancer cells via upregulation of carriers, such as the p-glycoprotein [22]. While the study of cancer adaption under selection pressure has classically involved studying cancer under treatment, it is important to consider that the mere fact that one renegade cell has managed to grow a tumor is a product of adaption to constitutive selective pressure present in any organ it tries to grow in. This process of outgrowing under natural constitutive selection involves the evasion of growth regulation via extrinsic cell death and immune surveillance [5]. Interestingly, in solid tumors, cell loss exceeds the rate of mitosis by more than 50% [23], suggesting high rates of intratumoral selection and an important role in cell death therein. Intriguingly, increased tumor growth coincides with an increase in oxidative stress through oncogene signaling, hypoxia, and ER stress, that requires cellular adaptation [24, 25]. Despite its well-established role in late-stage carcinogenesis and cancer treatment evasion and all evidence pointing towards an important role in tumor development, the dynamics underlying clonal selection in early-stage or even pretreatment carcinogenesis have been largely neglected to date. Insights from several cancer entities suggest that, apart from tissue-specific effects, early carcinogenesis may be driven by the same principles that apply to later stages, including oncogene signaling, changes in metabolism, an increase of proliferation rates, and immune evasion [26]. This suggests, that nascent tumor cells resort to the very same tools to adapt to selective pressure, yet the exact stimuli, influence, and sequence of these events remain to be clarified. Receptor-driven, extrinsic apoptosis, on the other hand, seems to be a double-edged sword regarding its involvement in the promotion or inhibition of (early) carcinogenesis. For instance, stimulation of the CD95 death receptor by CD95L is well-established as a mean of cytotoxic lymphocytes to kill tumor cells, and cancer cells of different entities may evade CD95L-mediated extrinsic apoptosis via decreased CD95 expression, upregulation of cFlip or downregulation of FADD (reviewed elsewhere [27]). While deletion of CD95 in ovarian and liver cancer models in vivo was shown to decrease tumor formation due to missing pro-proliferative signals emanating from CD95/CD95L [28], the very same in vivo data could be interpreted as the result of a lack of cell death selective pressure and therefore persistence and outgrowth of more benign clones. The same explanation could also partially account for less tumor progression in the absence of mouse TRAIL-Rs in KRAS-driven cancer models [29]. In addition to the potential role of extrinsically-triggered cell death as a tool for the selection-of-the-fittest clone, sublethal activation of effector caspase 3 by radiation has been shown to actively drive skin carcinogenesis by promoting genomic instability [30]. Along similar lines, activation of mitochondrial outer membrane permeability in a minority of mitochondria within cells (minority MOMP) can similarly drive sublethal caspase activation, which promotes genomic instability and cellular transformation [31] without the involvement of selection via cell death. Interestingly, these effects may also be caspase-independent, as a recent study has shown that a persister phenotype in lung adenocarcinoma and colorectal cancer cells caused by sublethal treatment with BH3 mimetics may be triggered by the integrated stress response [32], whereas the immunological implications of these findings remain to be elucidated [33]. Accordingly, clinical studies have revealed that a high apoptosis score does not translate into a benefit in overall survival in NSCLC patients [34] and may even be correlated with reduced overall survival in colorectal carcinoma [35]. Nevertheless, in the case of an effective MOMP triggered, a subsequent release of mitochondrial DNA, which is tightly regulated by the interplay between BAX and BAK [36], resulting in cGAS/STING1 activation, may ignite a strong inflammatory response, including type I interferon release alongside triggering apoptosis via release of cytochrome C [37]. Interestingly, recent studies suggest that radiation-induced caspase-signaling in tumor cells may restrict mitochondrial DNA-dependent inflammation [38, 39]. Intriguingly, type I interferon signaling leading to suboptimal induction of immunogenic cell death [40] seems to elicit a more aggressive, stem cell-like phenotype in mouse fibrosarcoma and mammary carcinoma cell lines, which is conveyed by the epigenetic regulator KDM1B [41]. Moreover, the dynamic network of interactions between pro- and antiapoptotic members of the BCL2 family have proven highly flexible and versatile in determining (cancer) cell fate by regulating intrinsic apoptosis susceptibility (reviewed elsewhere [42–45]).

Given the broad spectrum of cell death pathways known and based on our own work suggesting deregulation in multiple cell death pathways in treatment-naïve SCLC [46], it is certainly conceivable that cell death pathways may influence cancer cell selection in different ways and may be switched off during carcinogenesis in a hierarchical pattern. Consequently, exploring the “how” and “why” of early cancer cell adaption under selective pressure will greatly improve our understanding of carcinogenesis and may also provide novel targets for cancer diagnosis and therapy.

## The ever-diversifying portfolio of cell death pathways: survival by adaption

**Fig. 1:**
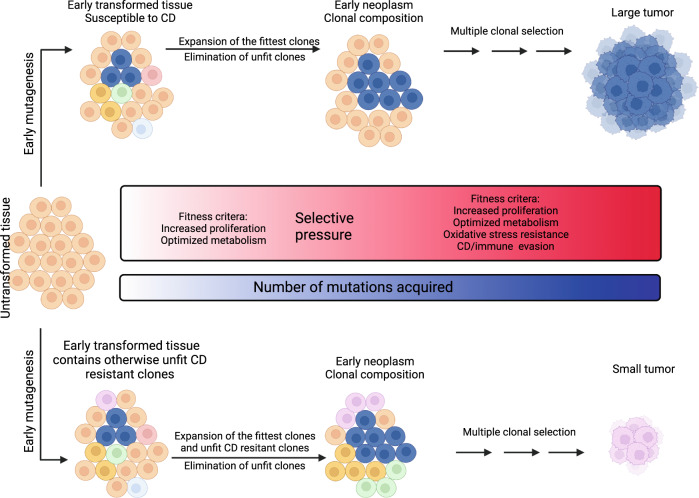
Cell death resistance in cancer evolution—a double-edged sword? During early tissue transformation, intercellular variations, e.g., in DNA replication and repair, may lead to early transformation through an accumulation of various (driver) mutations. These mutations, in turn, may increase clonal fitness by increasing proliferation and/or metabolism and hence enable single, advantageous clones to expand and form precursor lesions, while disadvantageous clones may be eliminated through various modes of cell death. Early neoplasm formation is accompanied by a steady increase in selective pressure through increases in oxidative stress and nutrient scarcity, which, in turn, fuels selection for even fitter clones carrying resistance to oxidative stress, cell death, and/or immune intervention, which may ultimately lead to tumor formation. However, when cell death resistance is induced during early tissue transformation, expansion of otherwise unfit clones may be enabled. This may ultimately lead to the formation of smaller and less invasive tumors through further rounds of selection. CD cell death. This figure was generated using fully licensed biorender.com.

In 1972, Kerr and colleagues coined the term apoptosis for a type of cell clearance program, that is morphologically distinct from necrosis [47]. Moreover, they recognized its role in healthy tissue maintenance and the possible role of apoptosis in neoplastic tissue homeostasis, thereby opening a whole new perspective on the (patho)physiological role of cell death. Since Kerr’s microscopy studies, the ever-growing arsenal of molecular biology methods has led to a much more detailed picture of the molecular machinery underlying programmed cell death. Apoptosis, which leads to cell shrinkage and blebbing while membrane integrity is maintained, and necroptosis, which is characterized by swelling of cytoplasm and organelles as well as permeabilization of cell membranes, are now regarded as extreme morphological manifestations within a plethora of different cell death programs, that is triggered by various types of cellular stress [48]. While apoptosis can either be triggered via ligand/receptor binding (extrinsic apoptosis) [49] or via mitochondrial damage or DNA damage (intrinsic apoptosis), necroptosis is mostly triggered by ligands of the TNF superfamily when caspase 8 is inactivated or absent [50]. Apoptotic cell death is executed via the activation of a caspase cascade, ultimately resulting in DNA fragmentation, necroptosis is inhibited by caspase activity and dependent upon RIPK1 kinase activity. As one would expect from this peculiar pathway mechanistic setup, aberrant necroptosis is unleashed upon caspase 8 deletion in the mouse [51, 52]. Thereby, extrinsic apoptosis and necroptosis are mechanistically closely intertwined, while cross-signaling to other cell death modalities is less well understood. Recently, ferroptosis was described as an iron-dependent type of regulated necrosis in which a chain reaction of membrane lipid peroxidation is thought to lead to cell death [53–56]. Importantly, ferroptosis seems to be independent of the molecular machinery driving apoptosis and necroptosis, yet its pathway effectors may be co-regulated with necroptosis pathway components in the context of T-cell attack. As an example, IFN-γ made by activated CD8 T-cells is known to induce expression of the core machinery of necroptosis [50] and was also recently described to upregulate ACSL4 [57], an essential component promoting cellular synthesis of a ferroptosis-prone lipidome known to be required for ferroptotic cell death [58]. In light of the strong selective environment posed by proliferating cancer cell populations competing for supplies under the surveillance of the immune system, one may wonder whether evasion of extrinsic cell death constitutes a selective advantage for overall tumor growth or whether loss of cell death may, in fact, be counterproductive for tumor evolution as a whole as it will sabotage principles of survival-of-the-fittest. While the exact role of the various cell death programs during tumorigenesis remains to be clarified, several keystones of this fascinatingly complex network have been implicated. For instance, loss-of-function mutations in p53 have long been regarded as one of the most important genetic events in oncogenic transformation due to the evasion of intrinsic apoptosis [5]. In recent years, however, a much more diverse role of p53 has become apparent. In fact, mutant p53 at the same time gains functions in promoting cellular migration [59], driving stress adaptation through changes in cancer cell metabolism [60], regulation of oxidative stress response mechanisms [61], as well as modulation of the tumor microenvironment. Thereby, whether a tumor loses p53 expression or expresses mutant p53 might affect its kinetic progression. Support for the hypothesis that steady rates of cell death may accelerate tumor evolution and, thereby, selection for the fittest clone stems from a study analyzing human lung adenocarcinoma patient data. In this study, a prognostic signature comprising 21 genes from apoptosis (including CASP9, FADD, and FAS), necroptosis (including MLKL and GSDME), and autophagy (including ATG5) was inferred from publicly available datasets. Interestingly, high expression of this signature was associated with increased stage of disease, lymph node involvement, and decreased overall survival, and evidence for enrichment of CD8 + T cells and macrophages was provided [62]. In light of the potentially ambivalent effects of cell death machinery on cancer cell fate and patient outcome, it is tempting to speculate, whether an early loss-of-function mutation in cell death pathway regulators may be disadvantageous as it may deprive cancer cell population from the capability to eliminate weaker clones yet later stage inactivation may be beneficial for the same clone as it would be able to grow unperturbed once nutrient/oxygen/space competitors are eliminated (Fig. 1).

## Cancer immune evasion: cell death pathway plasticity in time?

**Fig. 2:**
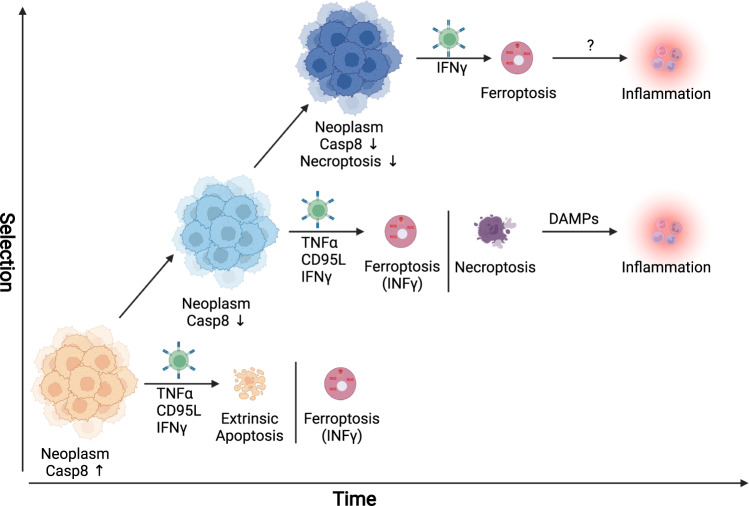
Time-dependent cell death pathway plasticity in cancer evolution. Neoplasms expressing caspase 8 are sensitive towards the induction of extrinsic apoptosis by cytotoxic T-cells secreting TNFα, CD95L, or TRAIL. In addition, IFNγ is secreted, which may also sensitize tumor cells to ferroptosis via lipid remodeling. Evasion of extrinsic apoptosis via loss-of-function/expression of caspase 8 is commonly observed in neoplasms, yet it will render tumor cells more sensitive to induction of necroptosis, which, in turn, may lead to an inflammatory response via the release of danger-associated molecular patterns (DAMPs). Finally, persistent selective pressure may result in downregulation/loss of necroptosis pathway components. Yet, this would leave IFNγ-induced sensitization towards ferroptosis intact. This figure was generated using fully licensed biorender.com.

While decades of research have shaped the concept that cancers can evade attack by adaptive immunity through the loss of expression of the antigen-presenting machinery, more recent evidence has shed light on the function of loss of regulated cell death in this process. This does not come as a surprise given that a major protein class released by effector cells of the immune system is TNF-superfamily ligands—the bona fide ligand family to induce extrinsic apoptosis and/or necroptosis. TNF-superfamily ligands, including TNF, CD95L, and TNF-related apoptosis-inducing ligand (TRAIL), can all induce extrinsic apoptosis, necroptosis, or NF-kB-mediated inflammatory gene induction as a primary or secondary signaling output depending on the ligand/receptor system engaged (reviewed [49, 63]). In all cases, expression of the protease caspase 8 is crucial to initiate extrinsic apoptosis. Interestingly, inactivating caspase 8 mutations were found to correlate with adaptive immune cytolytic activity in multiple cancer entities [64], supporting a concept wherein selective pressure via the immune system would lead to the inactivation of the extrinsic apoptosis pathway. In keeping with this argument, epigenetic downregulation of caspase 8 is a feature observed in many cancers. Yet interestingly, it is most frequently found in neuroendocrine cancer entities [46, 65, 66]; whether or not this is a feature of selection or an inherited trait from the cell-of-origin remains to be addressed. However, apart from its essential role in triggering extrinsic apoptosis induced by TNF-superfamily ligands, caspase 8 is now known to serve as a central switch between apoptosis, necroptosis, and pyroptosis [67, 68]. Thereby, loss of caspase 8 functionality can promote aberrant necroptosis as well as aberrant pyroptosis, two highly immunogenic types of cell death. Yet, cancers will evolve to select against immunogenicity and, therefore, likely lose the necroptosis and pyroptosis machinery as a consequence. Indeed, evidence from a CRISPR/Cas9 screen suggests that most factors eliminated in tumors in a manner dependent on adaptive immunity where components triggering necroptotic cell death besides components of the IFN-γ-pathway [69]. Apart from extrinsic apoptosis and necroptosis, interestingly, a recent study showed that CD8^+^ T-cells sensitize cancer cells to ferroptosis through IFN-γ-induced downregulation of xCT during immune checkpoint blockade [70], strongly suggesting ferroptosis to be a type of cell death naturally occurring in the tumor microenvironment which might be instrumental to immune cell-mediated “selection-of-the-fittest” even in the absence of immunotherapy. Along these lines, CD8 T-cells were shown to induce rewiring of the tumor cell lipidome in a manner making cell membranes more vulnerable to lipid peroxidation and ferroptosis [71]. Thereby, in the context of tumor evolution and repeated selective sweeps, we propose a concept of cell death pathway plasticity in time in which the inactivation of one cell death pathway will create collateral selective pressure via another (Fig. 2). Investigating hierarchies and dynamics of this process in tumor biology will likely yield a novel way of thinking about targeted cancer treatments not only as a strategy to kill but a strategy in which timing and dynamic cell death pathway regulation is crucial.

## Conclusions/outlook

From early neoplastic transformation to metastasis, cancer cells are continuously embedded into a highly competitive environment, either through resource scarcity, immune surveillance via death ligands or administration of therapeutic agents. Deregulation of cell death by loss-of-function mutations in tumor suppressor genes and immune evasion are well-established features of many, if not all, cancers. However, many of the current insights stem from rather late time points in the disease, as they mostly refer to studies in already diagnosed and/or treated patients. While this approach has yielded many valuable insights and has helped to improve treatment prognosis in many cases, the time span between early neoplastic transformation and detectable disease has been largely neglected. In order to ultimately aim at cancer prevention in human patients, studies investigating how cancer cell fate may be shaped during early carcinogenesis are much needed. Cell death evasion has long been considered a hallmark of cancer, but in light of the growing evidence of a more diverse role of the cell death machinery in cancer cell fate, it remains to be clarified, if cell death evasion may serve as a binary switch in early-onset carcinogenesis, or, whether certain amounts of cell death may in fact benefit tumor evolution in order to ensure selection for fitter clones with stronger proliferative/survival capabilities. Also, the plethora of possible perturbations in the various modes of cell death reported in a variety of cancers further raises the question, of whether these are part of continuous and rather transient processes or distinct selective events leading to clonal sweeps. Ferroptosis, a recently described mode of cell death, may be of particular interest in this regard, since it has been described as a more “primal” form of cell death, depending mostly on intracellular ROS and iron while showing a less complex intracellular network in comparison to other modes of cell death. While genetic evidence suggests that cancer cells can undergo various modes of regulated cell death and likely do so under constitutive selection pressure imposed by the immune system, future studies into a possible sequence of cell death pathway loss in cancer will ultimately reveal how regulated cell death pathways intersect in a physiological setting.

## Data Availability

Data sharing is not applicable to this article, as no datasets were generated or analyzed for this study.
